# Census Bulletin, No. 12

**Published:** 1890-12

**Authors:** 


CENSUS BULLETIN, NO. 12. Issued by Hon. Robert P. Porter, Superintendent of the Census.

Is a summary of the total population of the United States from which it appears that the total population is 62,480,540.

